# Probing the Fluxional Bonding Nature of Rapid Cope rearrangements in Bullvalene C_10_H_10_ and Its Analogs C_8_H_8_, C_9_H_10_, and C_8_BH_9_

**DOI:** 10.1038/s41598-019-53488-5

**Published:** 2019-11-19

**Authors:** Yuan-Yuan Ma, Miao Yan, Hai-Ru Li, Yan-Bo Wu, Xin-Xin Tian, Hai-Gang Lu, Si-Dian Li

**Affiliations:** Institute of Molecular Science, Shanxi University, Taiyuan, 030006 China

**Keywords:** Chemical physics, Electronic structure

## Abstract

Bullvalene C_10_H_10_ and its analogs semibullvalene C_8_H_8_, barbaralane C_9_H_10_, and 9-Borabarbaralane C_8_BH_9_ are prototypical fluxional molecules with rapid Cope rearrangements at finite temperatures. Detailed bonding analyses performed in this work reveal the existence of two fluxional π-bonds (2 2c-2e π → 2 3c-2e π → 2 2c-2e π) and one fluxional σ-bond (1 2c-2e σ → 1 4c-2e σ → 1 2c-2e σ) in their ground states and transition states, unveiling the universal π + σ double fluxional bonding nature of these fluctuating cage-like species. The highest occupied natural bond orbitals (HONBOs) turn out to be typical fluxional bonds dominating the dynamics of the systems. The ^13^C-NMR and ^1^H-NMR shielding tensors and chemical shifts of the model compound C_8_BH_9_ are computationally predicted to facilitate future experiments.

## Introduction

Chemical bond is the most fundamental and important concept in chemistry. Classical bonds include localized two-center-two-electron (2c-2e) bonds and delocalized multi-center-two-electron (mc-2e, m ≥ 3) bonds. Our group predicted the existence of fluxional σ- and π-bonds (FBs) in planar B_18_^2−^ and B_19_^−^, half-sandwich KB_18_^−^, tubular Ta@B_20_^−^, Ta@B_21_, and Ta@B_22_^+^, and cage-like B_39_^−^ in four recent papers^1–4^. Multicenter FBs in these fluctuating boron nanoclusters form and break constantly in concerted mechanisms at room temperatures. It is these FBs that facilitate the fluxional behaviors of these electron-deficient boron-based nanoclusters which possess energy barriers lower than the differences of the corresponding zero-point energy corrections. However, boron nanoclusters are known to be unstable in air and moisture and have hitherto been observed and characterized in gas phase only. Fluxional bonds in stable systems beyond boron which fluctuate rapidly and reversibly at finite temperatures remain to be explored in chemistry.

Prototypical fluxional molecules in organic chemistry include the norcaradiene-cycloheptatriene system, various annulenes, and homotropilidenes. Bridged homotropilidenes with degenerate valence-bond tautomerisms, such as the cage-like bullvalene C_10_H_10_, semibullvalene C_8_H_8_, barbaralane C_9_H_10_, and 9-borabarbaralane C_8_BH_9_, are of particular interest which exhibit reversible fluxionalities in rapid Cope rearrangements through a transition state with a bis-homoaromatic array of orbitals^5–17^. A topological analysis of experimental electron densities of the ground-state *C*_3*v*_ bullvalene was reported in 1996^18^. C_10_H_10_, C_8_H_8_, and C_9_H_10_ have the experimental free energy barriers of ΔG^≠^ = 12.8 kcal/mol at 100 °C, 5.5 kcal/mol at −143 °C, and 7.8 kcal/mol at −77 °C in NMR measurements, respectively^11^, while the model compound C_8_BH_9_ has the calculated ΔG^≠^ = 10.36 kcal/mol at 27 °C^17^. Semibullvalene C_8_H_8_ has proven to have the lowest fluxional energy barrier, fastest rearrangement rate, and lowest fluctuating temperature in the series^11^. Despite their differences in compositions and ground state structures, cage-like C_10_H_10_, C_8_H_8_, C_9_H_10_, and C_8_BH_9_ have similar transition state structures in rapid Cope rearrangements which have obvious multicenter bonding characteristics. However, the specific bonding patterns and fluxional bonding nature which facilitate the fluctuating behaviors of these intriguing molecules still remain unknown to date in both theory and experiments.

We aim to tackle the problem at first-principles theory level in this work. Detailed bonding analyses reveal a universal bonding pattern with two fluxional π-bonds and one fluxional σ-bond in the ground states and transition states of the C_10_H_10_, C_8_H_8_, C_9_H_10_, and C_8_BH_9_ series, unveiling the σ + π double fluxional bonding nature of these rapidly and reversibly fluctuating species. Their highest occupied natural bond orbitals appear to be typical fluxional bonds which dominate the fluxional behaviors of the systems in Cope rearrangements. We have also calculated the ^13^C-NMR and ^1^H-NMR shielding tensors and chemical shifts of the model compound C_8_BH_9_ to facilitate future NMR measurements.

### Theoretical Procedure

The ground-state (GS) and transition-state (TS) structures of the concerned species were fully optimized at density functional theory (DFT) level of PBE0^19^ with the basis sets of 6–311 + G(d)^20^. Frequency checks were performed to make sure all the optimized structures are true GMs or TSs. All the PBE0 structural optimizations and coupled cluster CCSD(T)^21–23^ single-point calculations in this work were performed using the Gaussian 09 package^24^. Detailed bonding analyses were performed on the concerned species using the adaptive natural density partitioning (AdNDP)^25–27^ method. The AdNDP approach recovers both the localized and delocalized bonding elements of the concerned systems and has been successfully applied to a wide range of nanoclusters and molecules^1–4,28–38^. Natural bonding orbital (NBO) analyses were performed utilizing the NBO 6.0 program^39^. The nuclear magnetic resonance (NMR) shielding tensors are calculated using the Continuous Set of Gauge Transformations (CSGT) method^40–42^ implemented in Gaussian09 program.

## Results and Discussions

### Structures and stabilities

**Figure 1:**
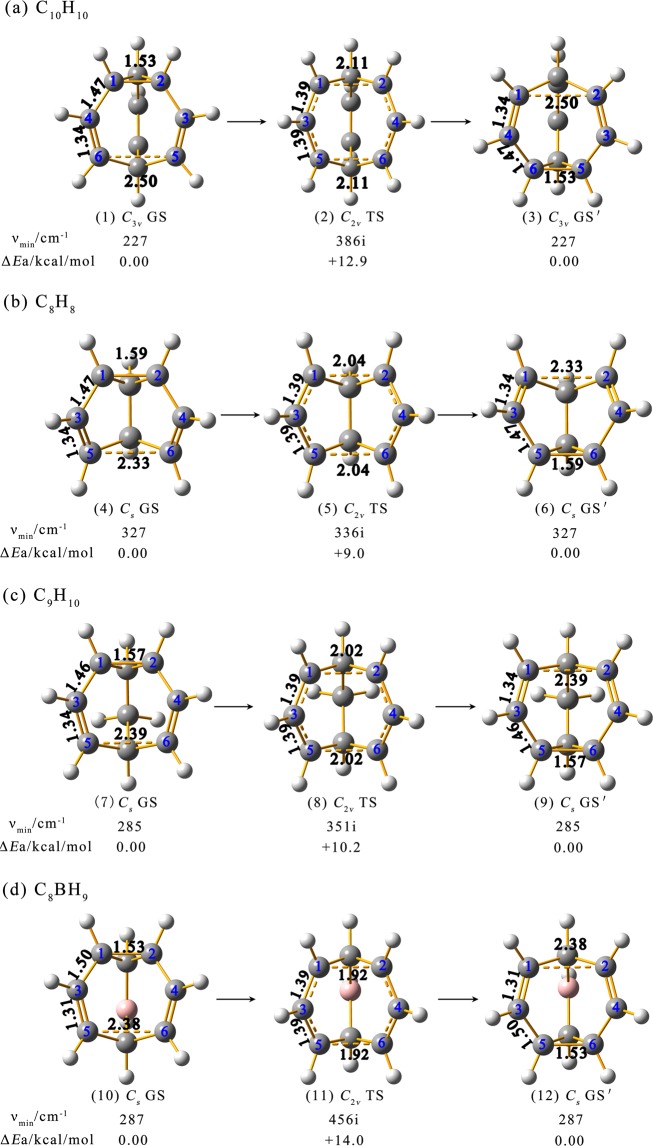
Optimized structures of the ground states (GSs/GSs′) and transition states (TSs) of (**a**) C_10_H_10_, (**b**) C_8_H_8_, (**c**) C_9_H_10_, and (**d**) C_8_BH_9_, with the lowest vibrational frequencies ν_min_ and relative energies Δ*E*_a_ indicated at PBE0 and CCSD(T) levels, respectively. Typical calculated C-C bond lengths are indicated in Å.

We start from the optimized structures of the GSs and TSs of concerned species first. As shown in Fig. 1, *C*_*3v*_ C_10_H_10_ (**1**), *C*_*s*_ C_8_H_8_ (**4**), *C*_*s*_ C_9_H_10_ (**7**), and *C*_*s*_ C_8_BH_9_ (**10**) as true minima of the systems possess cage-like structures with the lowest vibrational frequencies of 227, 303, 285, and 194 cm^−1^ at PBE0 level (Fig. 1), respectively. They all contain three C-C σ single bonds in the C_3_ triangle on the top and two C$\dddot{-}$C σ + π double bonds (C3$\dddot{-}$C5 and C4$\dddot{-}$C6) on the two long edges of the C_7_ heptagon in the front, with a mirror plane perpendicular to the paper surface. Their equivalent counterparts GSs′ C_10_H_10_ (**3**), *C*_*s*_ C_8_H_8_ (**6**), *C*_*s*_ C_9_H_10_ (**9**), and *C*_*s*_ C_8_BH_9_ (**12**) with a C_3_ triangle at the bottom are degenerate in energy with the GSs discussed above. Obviously, there exists no delocalized bonding interaction in the true minima GSs and GSs′ in which each carbon atom follows the octet rule. In contrast, the more open high-symmetry transition states *C*_*2v*_ C_10_H_10_ (**2**), *C*_*2v*_ C_8_H_8_ (**5**), *C*_*2v*_ C_9_H_10_ (**8**), and *C*_*2v*_ C_8_BH_9_ (**11**) with one imaginary frequency at −386i, −336i, −351i, and −456i cm^−1^ at PBE0, respectively, all feature two effective C$\dddot{-}$C$\dddot{-}$C multicenter π-bonding interactions over C1$\dddot{-}$C3$\dddot{-}$C5 and C2$\dddot{-}$C4$\dddot{-}$C6 units along the two long edges of the C_8_ octohedron in the front (with r_c1—c3_ = r_c2—c4_ = r_c3—c5_ = r_c4—c6_ = 1.39 Å). They lie 12.9, 9.0, 10.2, and 14.0 kcal/mol higher in energy than their ground states at CCSD(T)//PBE0 level at 298 K, respectively. Such energy barriers appear to be much higher than that previously reported in boron nanoclusters^1–4^. This can be qualitatively understood based on the fact that, due to its prototypical electron-deficiency, boron has the strong propensity to form delocalized σ and π bonds in highly reactive boron nanoclusters with extremely small energy barriers^1–4^, while the fluxional processes in **1**, **4**, **7**, and **10** possess much higher energy barriers because they involve the formations and breakages of C-C interactions in stable organic species. The C1-C2 single bond with r_c1-c2_ = 1.53~1.59 Å on the top in the *C*_*3v*_ or *C*_*s*_ GSs has been elongated to r_c1–c2_ = r_c5–c6_ = 1.92~2.04 Å in the *C*_*2v*_ TSs. The calculated C1--C2 and C5--C6 distances in the *C*_*2v*_ TSs appear to be about 0.5 Å longer than the sum of the single-bond covalent radii of two carbon atoms (r_c-c_ = 1.50 Å)^43^, indicating that the C1--C2 and C5--C6 interactions across the two long edges in *C*_*2v*_ TSs are much weaker than a usual C-C single bond. Such C--C distances also appear to be much longer than the C-C single bond (1.579 Å) observed between the two inverted carbon atoms in propellane^44,45^. The calculated distances of r_c3-c4_ = 2.9~3.2 Å in *C*_*2v*_ TSs (**2**, **5**, **8**, **11**) clearly show that there exists no bonding interaction between C3-C4. These transition states with two weak C--C interactions (C1--C2 and C5--C6) on the top and at the bottom of the C_8_ octahedron are at the critical points of Cope intramolecular rearrangements, where the original C1-C2 single σ-bond in the GS is to be broken while the C5-C6 σ-interaction in GS′ is to be formed simultaneously in the same process and vice versa. The six carbon atoms (1–6) in the front of the *C*_*2v*_ TSs can be divided into two equivalent groups weakly bonded together, with two effective parallel C$\dddot{-}$C$\dddot{-}$C multicenter bonds (C1$\dddot{-}$C3$\dddot{-}$C5 and C2$\dddot{-}$C4$\dddot{-}$C6) along the two long edges of the C_8_ octagon and two weak C--C interactions (C1--C2 and C5-C6) on the top and at the bottom between them. C_10_H_10_, C_8_H_8_, C_9_H_10_, and C_8_BH_9_ possess the calculated free energy barriers of ΔG^≠^ = 13.32 kcal/mol at 100 °C, 5.94 kcal/mol at −143 °C, 7.86 kcal/mol at −77 °C, and 10.84 kcal/mol at 27 °C at PBE0 level, respectively, well in line with the corresponding values previously reported for these species at finite temperatures^11,17^.

### AdNDP bonding analyses

**Figure 2:**
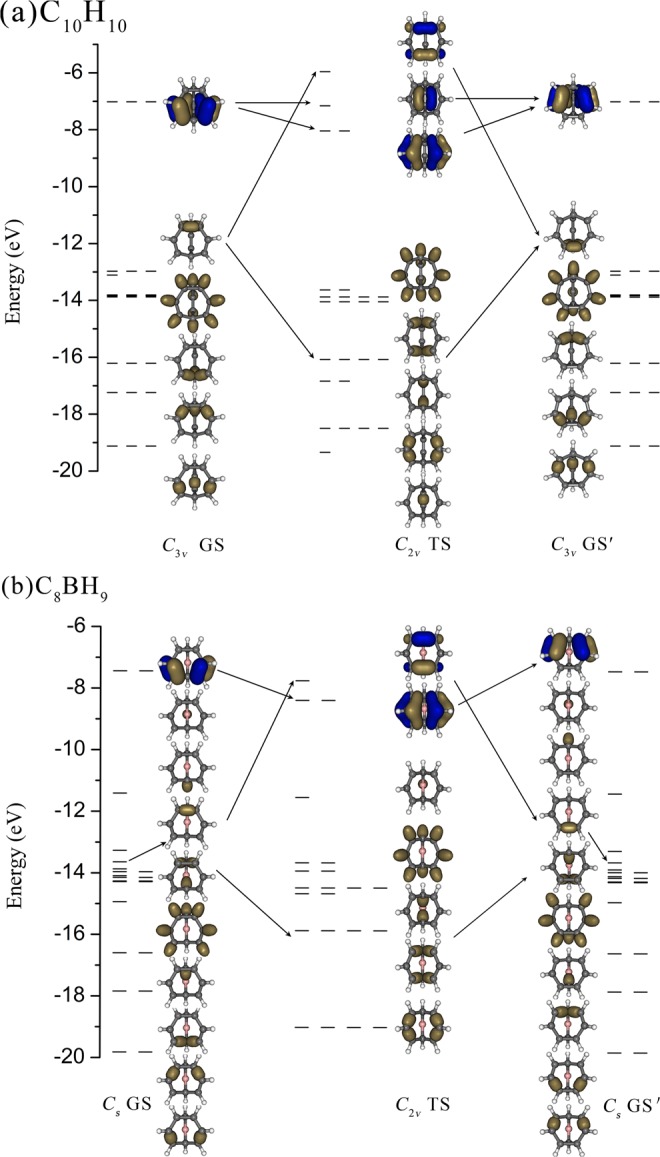
AdNDP natural bond orbital energy levels and bonding patterns of the ground states (GSs/GSs′) and transition states (TSs) of (**a**) C_10_H_10_ and (**b**) C_8_BH_9_ at PBE0/6-311 + G (**d**) level, with the two fluxional π-bonds and one fluxional σ-bond interlinked by arrowed lines from GS, TS, to GS′.

**Figure 3:**
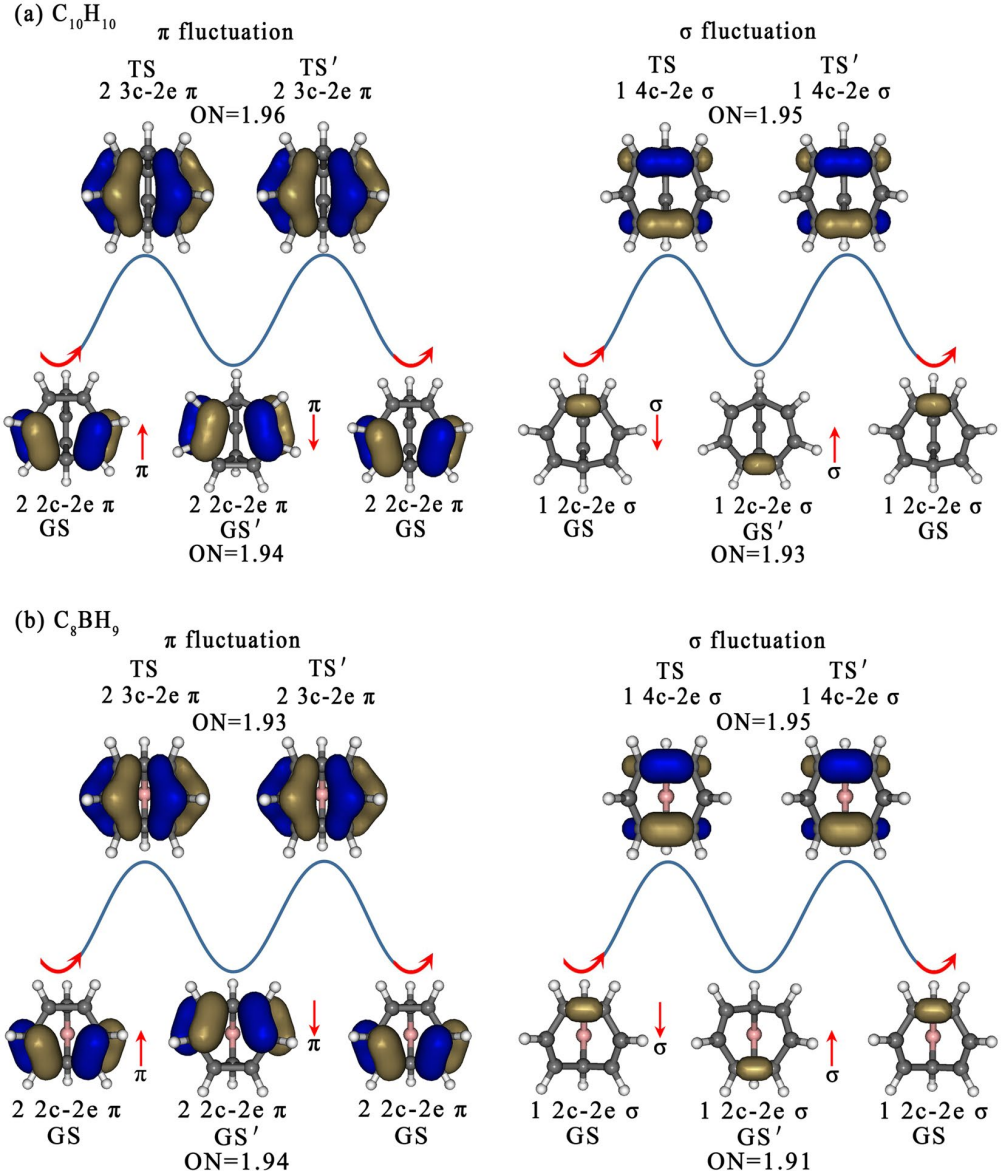
π- and σ-bonding fluctuations of (**a**) C_10_H_10_ and (**b**) C_8_BH_9_ in a full circle GS → TS → GS′ → TS′ → GS), with the two fluxional π-bonds and one fluxional σ-bond fluctuating up and down in opposite directions indicated by red arrows. The ON values represent the calculated occupation numbers of corresponding bonds.

The calculated AdNDP natural bond orbital energy levels of the GSs/GSs′ and TSs of C_10_H_10_ and C_8_BH_9_ are comparatively shown in Fig. 2, with that of C_8_H_8_ and C_9_H_10_ depicted in Fig. S2. These natural bond orbital energy levels reveal the bonding patterns of the concerned molecules clearly, exhibit the symmetries of the concerned species perfectly, and show the relative energies of the symmetrically distributed chemical bonds of the systems directly. The localized AdNDP natural bond orbitals have the advantage over the delocalized canonical molecular orbitals (CMOs) in providing a pictorial representation of the relative energies of the concerned chemical bonds and their electron density distributions in space, well in line with chemical intuitions. As anticipated, *C*_*3v*_ GM C_10_H_10_ (**1**) possesses 3 equivalent 2c-2e C-C π bonds with the occupation numbers of ON = 1.94 along the three long edges as its highest occupied natural bond orbitals (HONBOs) and 3 equivalent 2c-2e C-C σ bonds with ON = 1.93 on the top C_3_ triangle as the second highest occupied natural bond orbitals (HONBO-1), together with the remaining 9 2c-2e C-C σ bonds and 10 2c-2e C-H σ bonds to form the GS in an overall bonding symmetry of C_*3v*_ (Fig. 3a). From C_*3v*_ GS to *C*_*2v*_ TS, two π-HONBOs of the GS in the front (2 2c-2e π bonds over C3-C5 and C4-C6) are converted into 2 3c-2e π bonds as HONBO-2 of the *C*_*2v*_ TS over C1$\dddot{-}$C3$\dddot{-}$C5 and C2$\dddot{-}$C4$\dddot{-}$C6 with ON = 1.96 on the two long edges, one σ-HONBO-1 of the GS in the front (1 2c-2e σ bond on C1-C2 on the top C_3_ triangle) is transferred into 1 4c-2e σ-bond with ON = 1.95, the HONBO of the TS, which is evenly distributed on C1–C4 and C5–C6 with obvious bonding/antibonding characteristics, while the remaining 1 2c-2e π bond, 11 2c-2e C-C σ bonds, and 10 C-H σ bonds remain basically unchanged. The delocalized 4c-2e σ-bond is a σ + π mixture between two sets of titled p_z_-p_z_ pair interactions, with the major contribution from a head-to-head σ-overlap and minor contribution from a shoulder-by-shoulder π-overlap. An opposite process occurs from *C*_*2v*_ TS to the second minimum *C*_*3v*_ GS′. Thus, as clearly shown in Fig. 3a, in a full fluxional process C_*3v*_ GS → *C*_*2v*_ TS → C_*3v*_ GS′ → *C*_*2v*_ TS′ → C_*3v*_ GS, C_10_H_10_ undergoes a π-fluctuation of 2 2c-2e π (HONBOs) → 2 3c-2e π (HONBO-2) → 2 2c-2e π′ (HONBOs) → 2 3c-2e π′(HONBO-2) → 2 2c-2e π (HONBOs) and a σ-fluctuation of 1 2c-2e σ (HONBO-1) → 1 4c-2e σ (HONBO) → 1 2c-2e σ′ (HONBO-1) → 1 4c-2e σ′ (HONBO) → 1 2c-2e σ (HONBO-1) simultaneously in a concerted mechanism. Such a bonding fluctuation process occurs randomly in three equivalent directions perpendicular to the three equivalent C_7_ heptagons around the *C*_*3*_ molecular axis in both *C*_*3v*_ GS and GS′, generating 10!/3 equivalent isomers (~1.2 million) in total for C_10_H_10_, making all the ten H atoms magnetically equivalent with one signal observed in NMR measurements above 100 °C^15^.

Similarly, a π-fluctuation of 2 2c-2e π (HONBOs) → 2 3c-2e π (HONBO-1) → 2 2c-2e π′(HONBOs) → 2 3c-2e π′ (HONBO-1) → 2 2c-2e π (HONBOs) and a σ-fluctuation of 1 2c-2e σ (HONBO-3) → 1 4c-2e σ (HONBO) → 1 2c-2e σ′ (HONBO-3) → 1 4c-2e σ′ (HONBO) → 1 2c-2e σ (HONBO-3) occur in C_8_BH_9_ in a full fluxional circle (Fig. 3b). The two AdNDP π bonds and one AdNDP σ bond of C_10_H_10_ and C_8_BH_9_ have their origin from the two CMO π orbitals and one CMO σ orbital in them in both the ground state and transition state, as shown in Fig. S1, well supporting the AdNDP bonding patterns presented in Figs. 2 and 3. As shown in Fig. S3, C_8_H_8_ and C_9_H_10_ also exhibit two similar fluxional π bonds (2 2c-2e π → 2 3c-2e π → 2 2c-2e π) and one fluxional σ-bond (1 2c-2e σ → 1 4c-2e σ → 1 2c-2e σ) in reversible Cope rearrangements. However, different from *C*_*3v*_ C_10_H_10_ which possesses three equivalent fluctuating directions, *C*_*s*_ C_8_H_8_, *C*_*s*_ C_9_H_10_, and *C*_*s*_ C_8_BH_9_ can only fluctuate in one direction in the front C_7_ heptagon perpendicular to the mirror plane to form two equivalent isomers (Fig. 1). In particular, it is noticed that the HONBOs which have the highest natural bond orbital energies and highest relative reactivity in both the GSs and TSs in Cope intramolecular rearrangements are typical fluxional bonds which dominate the fluxional behaviors of the concerned systems (Figs. 2, 3 and S3). The *C*_*3v*_/*C*_*s*_ GSs all have π-HONBOs, while the *C*_*2v*_ TSs possess σ-HONBOs (Fig. 2 and S2). Such a natural bond orbital energy order renders low stability and high reactivity to the *C*_*2v*_ TSs.

The calculated electron numbers from the involved C atoms to the fluxional 3c-2e π-bonds and fluxional 4c-2e σ-bond in Table 1 indicate that, in the *C*_*2v*_ transition states of these fluxional molecules, the two central C atoms (C3 and C4) on the two long edges each contribute one electron to the respective 3c-2e π bond over C1$\dddot{-}$C3$\dddot{-}$C5 or C2$\dddot{-}$C4$\dddot{-}$C6, while the four C atoms on the top (C1 and C2) and at the bottom (C5 and C6) each contribute approximately half an electron. Meanwhile, the four C atoms on the top (C1 and C2) and at the bottom (C5 and C6) each contribute half an electron to the delocalized 4c-2e σ-bond, forming a half σ-bond on the top over C1--C2 and a half σ-bond at the bottom over C5--C6. The two separated half σ-bonds over C1-C2 and C5-C6 are antibonding in nature. As shown in Figs. 3a,b and S3a,b and Video S1, the two fluxional π bonds and one fluxional σ-bond in each species fluctuate up and down continuously and reversibly in opposite directions to keep the balance of σ- and π-bond densities in the fluctuating molecules.

**Table 1:** Calculated electron numbers from specific carbon atoms (1–6) contributed to the respective fluxional 3c-2e π-bonds and fluxional 4c-2e σ-bond in the transition states *C*_*2v*_ C_10_H_10_ (**2**) and *C*_*2v*_ C_8_BH_9_ (**11**) at PBE0/6-311 + G(d) level.

TSs	Atoms	3c-2e π-bonds	4c-2e σ-bond
*C*_*2v*_ C_10_H_10_	C1(C2)	0.47	0.49
	C3(C4)	1.02	—
	C5(C6)	0.47	0.49
*C*_*2v*_ C_8_BH_9_	C1(C2)	0.45	0.49
	C3(C4)	1.03	—
	C5(C6)	0.45	0.49

The calculated NBO bond orders of the *C*_*2v*_ transition states of these molecules in Fig. S4 also well support the bonding patterns presented above, with C1--C2, C1$\dddot{-}$C3, C3$\dddot{-}$C5, and C5--C6 interactions possessing the bond orders of 0.35, 1.46, 1.46, and 0.35 in *C*_*2v*_ C_10_H_10_ and 0.43, 1.43, 1.43, and 0.43 in *C*_*2v*_ C_8_BH_9_, respectively. The simultaneous formation of both the 2 3c-2e fluxional π-bonds and 1 4c-2e fluxional σ-bond in *C*_*2v*_ TS is a natural requirement to convert GS and GS′ backward and forward in a continuously and reversibly fluctuating process (Figs. 3 and S3).

### NMR shielding constants

NMR has proved to be a powerful tool for the determination of the energy barriers and rate constants of molecules with fluxional bonds in rapid Cope rearrangements^11^. The calculated absolute ^13^C- and ^1^H-NMR shielding tensors δ and chemical shifts ∆δ relative to tetramethylsilane (TMS) are tabulated for *C*_*3v*_ C_10_H_10_ (**1**) and *C*_*s*_ C_8_BH_9_ (**10**) in Table 2 in ppm. Our calculated ^13^C and ^1^H chemical shifts (∆δ) of bullvalene C_10_H_10_ agree well with that measured in NMR experiments at −59.9 °C and −59.2 °C, respectively^15^. (Table 2). The predicted ^13^C-NMR spectrum of the GM *C*_*s*_ C_8_BH_9_ at 298 K exhibits five kinds of C atoms with the absolute magnetic shielding tensors of δ = 55.23, 60.28, 144.00, 144.77, and 160.20 ppm in the intensity ratios of 2:2:2:1:1, respectively, while the corresponding ^1^H-NMR shielding tensors are calculated to be at δ = 23.04, 25.49, 25.75, 27.85, 28.41, and 29.19 ppm in the ratios of 1:2:2:1:2:1 (with ^1^H-B having the lowest ^1^H-NMR shielding constant). The B atom in *C*_*s*_ C_8_BH_9_ has the calculated ^11^B-NMR shielding tensor of δ = 24.41 ppm.

**Table 2:** Calculated absolute ^13^C-NMR and ^1^H-NMR shielding tensors δ and chemical shifts Δδ with TMS as internal reference in *C*_*3v*_ C_10_H_10_ (**1**) and *C*_*s*_ C_8_BH_9_ (**10**) at PBE0/6-311 + G(d) level.

	^13^C-NMR δ/ppm	^13^C-NMR Δδ/ppm	^1^H-NMR δ/ppm	^1^H-NMR Δδ/ppm
*C*_*3v*_ C_10_H_10_ (1)	54.62 (3)	130.39 (3) [128.5]	25.33 (3)	5.07 (3) [5.70]
	54.72 (3)	130.29 (3) [128.3]	25.48 (3)	4.92 (3) [5.62]
	152.27 (1)	32.75 (1) [31.0]	28.99(1)	1.41 (1) [2.13]
	162.74 (3)	22.28 (3) [21.0]	29.02(3)	1.38(3) [2.07]
*C*_*s*_ C_8_BH_9_ (10) (298 K)	55.23 (2)	129.78 (2)	23.04 (1)	7.36 (1)
	60.28 (2)	124.73 (2)	25.49 (2)	4.92 (2)
	144.00 (2)	41.01 (2)	25.75 (2)	4.65 (2)
	144.77 (1)	40.24 (1)	27.85 (1)	2.56 (1)
	160.20 (1)	24.82 (1)	28.41 (2)	1.99(2)
			29.19 (1)	1.22 (1)

The experimental ^13^C and ^1^H chemical shifts (∆δ) of C_10_H_10_ (**1**) at −59.9 °C and −59.2 °C are cited in square brackets for comparison, respectively^15^.

In summary, detailed AdNDP bonding analyses performed in this work reveal the existence of two fluxional π-bonds and one fluxional σ-bond in bullvalene C_10_H_10_ and its analogs C_8_H_8_, C_9_H_10_, and C_8_BH_9_. These fluxional bonds form and break constantly and reversibly in rapid Cope rearrangements at finite temperatures. Such a universal π + σ double fluxional bonding pattern reflects both the structural characteristics and fluxional bonding nature of these rapidly fluctuating species. Their HONBOs with the highest relative energies and reactivity belong to typical fluxional bonds which dominate the dynamics of the systems. The fluxional behaviors of these organic molecules are different in nature from that of the classical fluxional molecules like iron pentacarbonyl (Fe(CO)_5_), phosphorus pentafluoride (PF_5_), and dimethylformamide (CH_3_)_2_NC(O)H which undergo Berry pseudo-rotations^46–51^ via bond bending, swing, or stretching of localized 2c-2e σ bonds without the breaks or formations of chemical bonds involved in the fluxional process. Explorations of fluxional bonds in more complicated intramolecular rearrangements known in chemistry are currently underway.

## Supplementary information

Supplementary Information
