# Supplementary material for: Probing the Fluxional Bonding Nature of Rapid Cope rearrangements in Bullvalene C10H10 and Its Analogs C8H8, C9H10, and C8BH9
Source: Sci Rep. 2019 Nov 19;9:17074. doi: 10.1038/s41598-019-53488-5 (PMC6864245; doi:10.1038/s41598-019-53488-5)
Supplement: Supplementary file 1 — Supplementary Information [file 41598_2019_53488_MOESM1_ESM.docx]

**Probing the Fluxional Bonding Nature of Rapid Cope rearrangements in Bullvalene C_10_H_10_ and Its Analogs C_8_H_8_, C_9_H_10_, and C_8_BH_9_**

**Yuan-Yuan Ma, Miao Yan, Hai-Ru Li, Yan-Bo Wu, Xin-Xin Tian, Hai-Gang Lu, and Si-Dian Li*^1^**

Correspondence to: Si-Dian Li (E-mail: [lisidian@sxu.edu.cn](mailto:lisidian@sxu.edu.cn))

**Figure S1.** Comparison of the two AdNDP π bonds and one AdNDP σ bond of C_10_H_10_ (a) and C_8_BH_9_ (b) and the two CMO π orbitals and one σ orbital in them in both the ground state (GS) and transition state (TS).

**Figure S2.** AdNDP orbital energy levels of (a) C_8_H_8_ and (b) C_9_H_10_.

**Figure S3.** Bonding fluctuations of (a) C_8_H_8_ and (b) C_9_H_10_.

**Figure S4.** NBO bond orders of the *C*_2_*_v_* TSs of (a) C_10_H_10_, (b) C_8_H_8_, (c) C_9_H_10_, and (d) C_8_BH_9_.

**Table S1** Atomic orbital contributions to the two fluxional 3c-2e π-bonds and one fluxional 4c-2e σ-bond in the transition states of C_8_H_8_ and C_9_H_10_.

**Table S2** Optimized coordinates of **1-12** at PBE0/6-311+G(d).


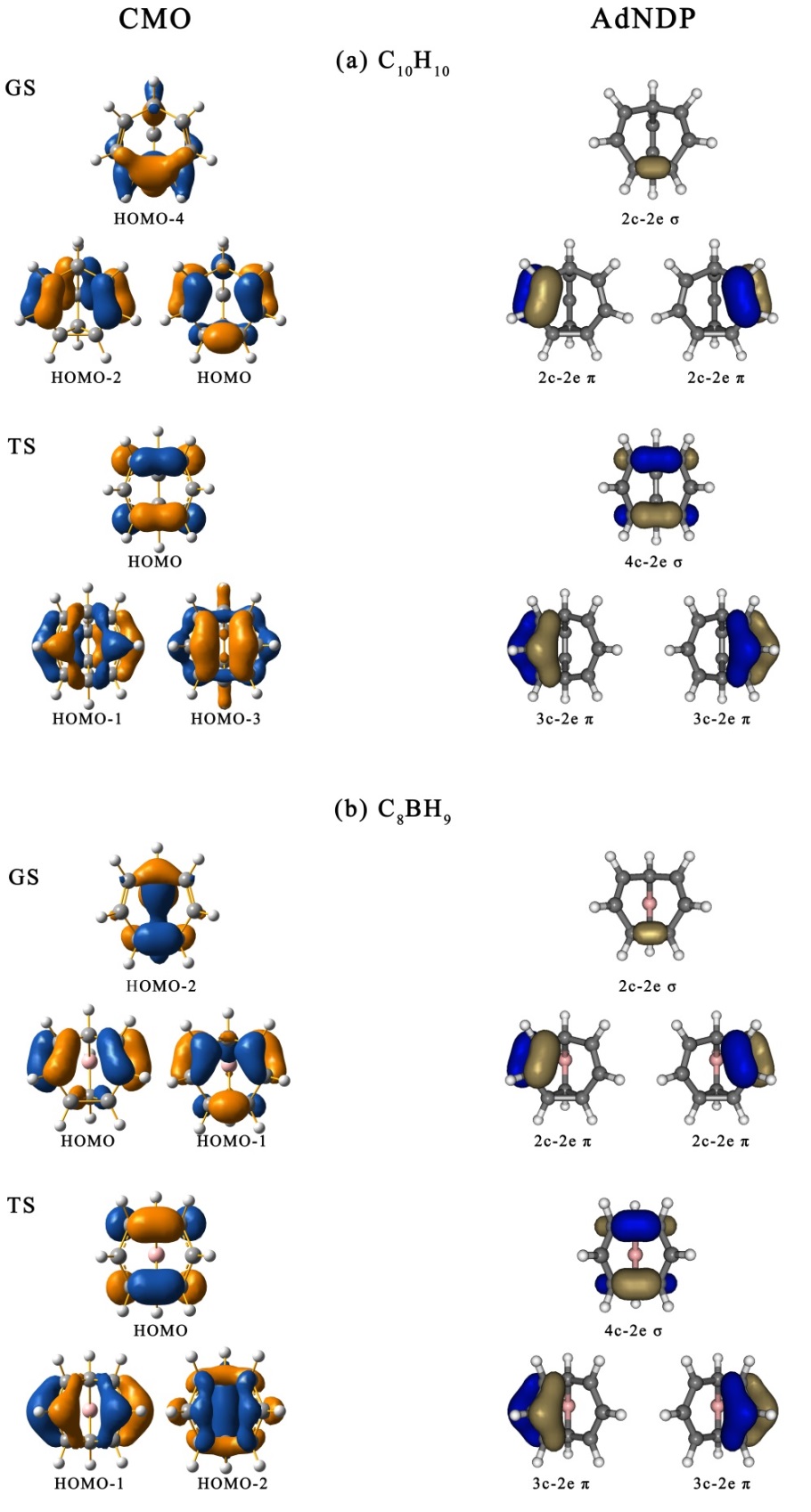
**Figure S1.** Comparison of the two AdNDP π bonds and one AdNDP σ bond of C_10_H_10_ (a) and C_8_BH_9_ (b) and the two CMO π orbitals and one σ orbital in them in both the ground state (GS) and transition state (TS).

**Figure S2.** AdNDP orbital energy levels of (a) C_8_H_8_ and (b) C_9_H_10_.


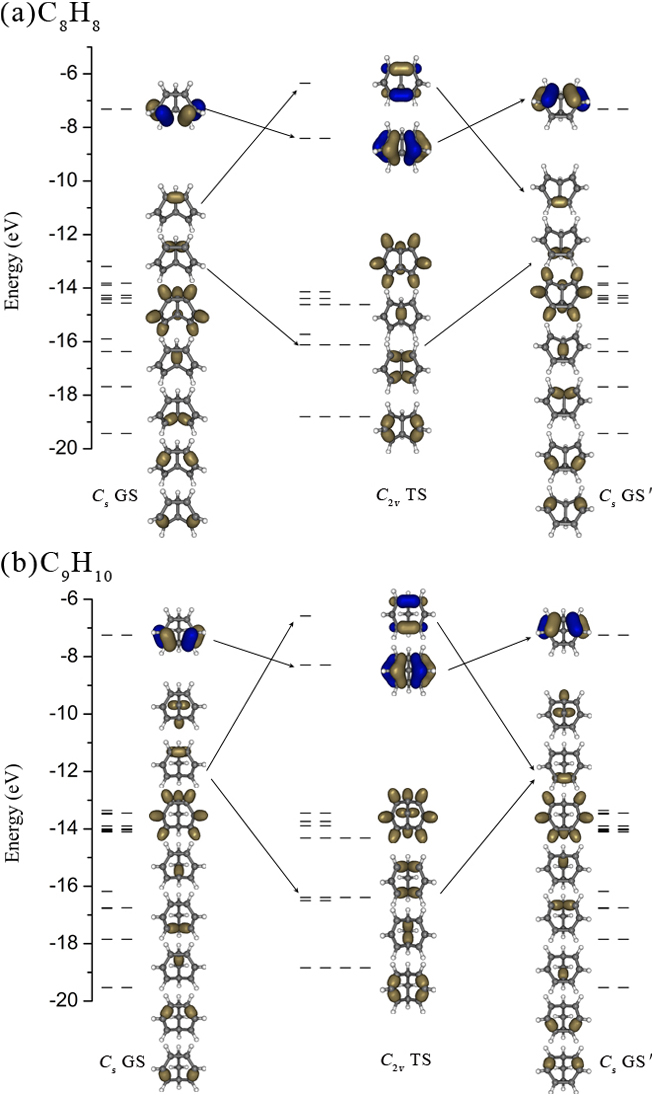


**Figure S3.** π- and σ-bonding fluctuations of (a) C_8_H_8_ and (b) C_9_H_10_ in a full circle GS→TS→GS′→TS′→GS), with the two fluxional π-bonds and one fluxional σ-bond fluctuating up and down in opposite directions indicated by red arrows. The ON values represent the calculated occupation numbers of corresponding bonds.


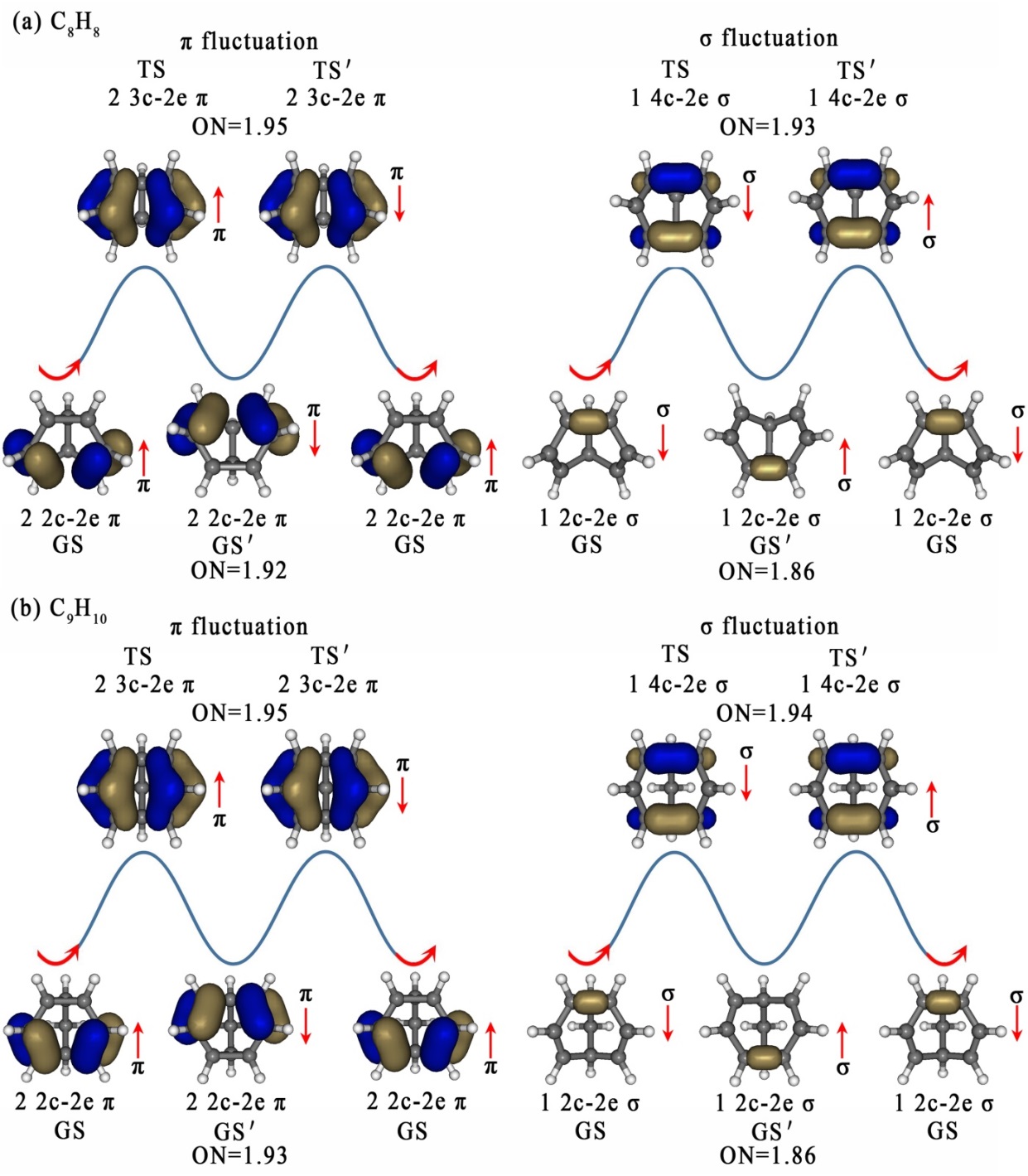


**Figure S4.** NBO bond orders of the *C*_2_*_v_* TSs of (a) C_10_H_10_, (b) C_8_H_8_, (c) C_9_H_10_, and (d) C_8_BH_9._


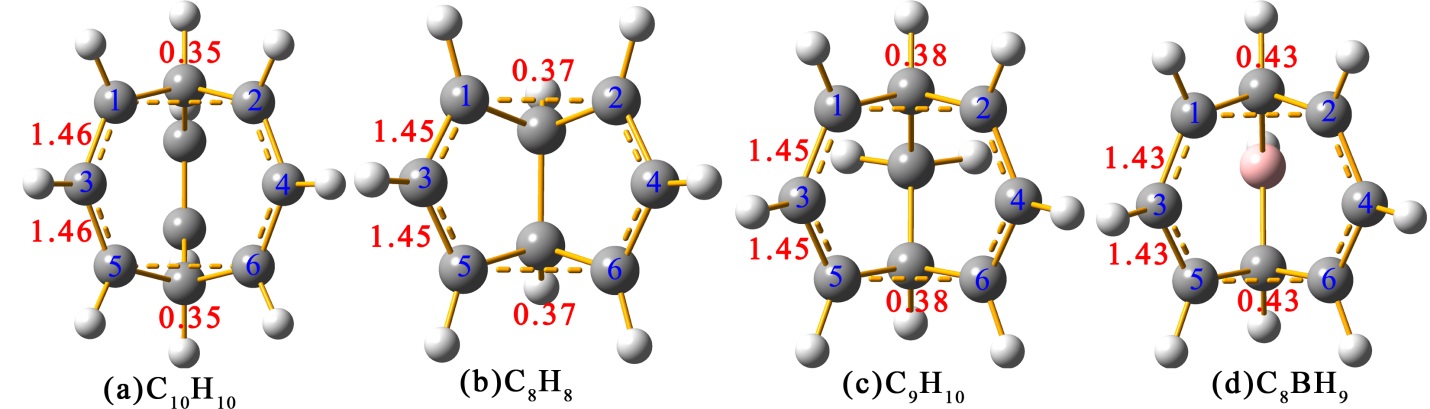


**Table S1** Calculated electron numbers from specific carbon atoms (1-6) contributed to the respective fluxional 3c-2e π-bonds and fluxional 4c-2e σ-bond in the transition states *C_2v_* C_8_H_8_ (**5**) and *C_2v_* C_9_H_10_ (**8**) at PBE0/6-311+G(d) level.

| TSs | Atoms | 2 3c-2e π-bonds | 1 4c-2e σ-bond |
| --- | --- | --- | --- |
| *C_2v_* C_8_H_8_ | C1 and C2 | 0.47 | 0.48 |
|  | C3 and C4 | 1.00 | - |
|  | C5 and C6 | 0.47 | 0.48 |
| *C_2v_* C_9_H_10_ | C1 and C2 | 0.46 | 0.49 |
|  | C3 and C4 | 1.02 | - |
|  | C5 and C6 | 0.46 | 0.49 |

**Table S2** Optimized coordinates of **1-12** at PBE/6-311+G(d).

GS *C_3v_* C_10_H_10_ (**1**)

C 0.00000000 0.88791628 -1.37219485

C -0.76895805 -0.44395814 -1.37219485

C -1.56320452 -0.90251655 -0.21515548

C 0.00000000 1.80503310 -0.21515548

C -1.25205749 -0.72287573 1.08061862

C -0.00000000 1.44575146 1.08061862

C 0.00000000 0.00000000 1.55532903

H 0.00000000 1.39179292 -2.33554777

H -1.20532803 -0.69589646 -2.33554777

H -2.48243067 -1.43323201 -0.46018878

H 0.00000000 2.86646403 -0.46018878

H -1.92581246 -1.11186834 1.84228940

H -0.00000000 2.22373668 1.84228940

H 0.00000000 0.00000000 2.64952003

C 1.56320452 -0.90251655 -0.21515548

C 0.76895805 -0.44395814 -1.37219485

C 1.25205749 -0.72287573 1.08061862

H 2.48243067 -1.43323201 -0.46018878

H 1.20532803 -0.69589646 -2.33554777

H 1.92581246 -1.11186834 1.84228940

TS *C*_2_*_v_* C_10_H_10_ (**2**)

C 1.05259311 -1.24860773 -0.56629423

C -1.05259311 -1.24860773 -0.56629423

C -1.51371209 0.00000000 -0.98044195

C 1.51371209 0.00000000 -0.98044195

C -1.05259311 1.24860773 -0.56629423

C 1.05259311 1.24860773 -0.56629423

C 0.00000000 1.50897887 0.47836811

H 1.43498349 -2.12557893 -1.08241634

H -1.43498349 -2.12557893 -1.08241634

H -2.22703414 0.00000000 -1.80343391

H 2.22703414 0.00000000 -1.80343391

H -1.43498349 2.12557893 -1.08241634

H 1.43498349 2.12557893 -1.08241634

H 0.00000000 2.56667484 0.75009722

C 0.00000000 -0.66811926 1.72532889

C 0.00000000 -1.50897887 0.47836811

C -0.00000000 0.66811926 1.72532889

H 0.00000000 -1.20270636 2.67416984

H 0.00000000 -2.56667484 0.75009722

H -0.00000000 1.20270636 2.67416984

GS *C_3v_* C_10_H_10_ (**3**)

C 1.25203573 -0.72286316 1.08060138

C -1.25203573 -0.72286316 1.08060138

C -1.56328389 -0.90256238 -0.21517270

C 1.56328389 -0.90256238 -0.21517270

C -0.76898055 -0.44397113 -1.37216331

C 0.76898055 -0.44397113 -1.37216331

C 0.00000000 0.88794226 -1.37216331

H 1.92582996 -1.11187844 1.84222255

H -1.92582996 -1.11187844 1.84222255

H -2.48251867 -1.43328282 -0.46014715

H 2.48251867 -1.43328282 -0.46014715

H -1.20535914 -0.69591442 -2.33551852

H 1.20535914 -0.69591442 -2.33551852

H 0.00000000 1.39182884 -2.33551852

C -0.00000000 1.44572633 1.08060138

C 0.00000000 0.00000000 1.55533407

C 0.00000000 1.80512475 -0.21517270

H -0.00000000 2.22375689 1.84222255

H 0.00000000 0.00000000 2.64952907

H 0.00000000 2.86656565 -0.46014715

GS *C_s_* C_8_H_8_ (**4**)

C -0.12424253 -1.10787477 1.16697600

C -0.68471019 0.05247777 1.53946900

C -0.68471019 0.05247777 -1.53946900

C -0.12424253 -1.10787477 -1.16697600

H -0.43225060 -2.09422263 1.49944100

H -1.50449420 0.15501533 2.24285200

H -1.50449420 0.15501533 -2.24285200

H -0.43225060 -2.09422263 -1.49944100

C -0.10314558 1.17693516 -0.79702900

C -0.10314558 1.17693516 0.79702900

C 1.04174938 0.63902476 0.00000000

H 2.03090322 1.08491130 0.00000000

C 0.82493556 -0.88947385 0.00000000

H 1.72744097 -1.50473596 0.00000000

H -0.06989227 2.17123796 -1.23001200

H -0.06989227 2.17123796 1.23001200

TS *C*_2_*_v_* C_8_H_8_ (**5**)

C -1.13925500 1.01890900 -0.10097511

C 0.00000000 1.55893600 -0.67813011

C 0.00000000 -1.55893600 -0.67813011

C -1.13925500 -1.01890900 -0.10097511

H -2.16168000 1.29266800 -0.32752111

H 0.00000000 2.23212600 -1.52818411

H 0.00000000 -2.23212600 -1.52818411

H -2.16168000 -1.29266800 -0.32752111

C 1.13925500 -1.01890900 -0.10097511

C 1.13925500 1.01890900 -0.10097511

C 0.77591100 -0.00000000 0.92914189

H 1.29363600 -0.00000000 1.88885689

C -0.77591100 -0.00000000 0.92914189

H -1.29363600 -0.00000000 1.88885689

H 2.16168000 -1.29266800 -0.32752111

H 2.16168000 1.29266800 -0.32752111

GS *C_s_* C_8_H_8_ (**6**)

C 0.10319035 1.17698279 0.79711700

C 0.68504874 0.05247809 1.53828400

C 0.68504874 0.05247809 -1.53828400

C 0.10319035 1.17698279 -0.79711700

H 0.06987935 2.17134326 1.22993400

H 1.50591444 0.15401308 2.24056100

H 1.50591444 0.15401308 -2.24056100

H 0.06987935 2.17134326 -1.22993400

C 0.12433156 -1.10788170 -1.16631200

C 0.12433156 -1.10788170 1.16631200

C -0.82607336 -0.88963404 0.00000000

H -1.72923651 -1.50369267 0.00000000

C -1.04194130 0.63900079 0.00000000

H -2.03039136 1.08644805 0.00000000

H 0.43264015 -2.09430932 -1.49825300

H 0.43264015 -2.09430932 1.49825300

GS *C_s_* C_9_H_10_ (**7**)

C -0.45844810 1.32627415 0.00000000

C 0.44382246 1.13376310 0.78733000

C 0.81979857 -0.10820951 1.52765500

C -1.56604482 0.25713832 0.00000000

C 0.31876617 -1.26808897 1.04373300

C -0.95044136 -1.11516730 0.00000000

H -0.90222472 2.32530540 0.00000000

H 0.84265349 2.00236557 1.30425900

H 1.53158080 -0.27754656 2.33155500

H 0.52170340 -2.24456654 1.47152800

H -1.63315683 -1.96009904 0.00000000

C 0.81979857 -0.10820951 -1.52765500

C 0.44382246 1.13376310 -0.78733000

C 0.31876617 -1.26808897 -1.04373300

H 1.53158080 -0.27754656 -2.33155500

H 0.84265349 2.00236557 -1.30425900

H 0.52170340 -2.24456654 -1.47152800

H -2.19776733 0.38762122 -0.88581700

H -2.19776733 0.38762122 0.88581700

TS *C*_2_*_v_* C_9_H_10_ (**8**)

C -1.23997700 0.00000000 0.72454881

C -1.20688200 1.01206700 -0.36711519

C 0.00000000 1.50639600 -0.84013619

C 0.00000000 0.00000000 1.60118081

C 1.20688200 1.01206700 -0.36711519

C 1.23997700 0.00000000 0.72454881

H -2.16777800 0.00000000 1.29829681

H -2.14722600 1.35860600 -0.78246119

H 0.00000000 2.19217200 -1.68269819

H 2.14722600 1.35860600 -0.78246119

H 2.16777800 0.00000000 1.29829681

C 0.00000000 -1.50639600 -0.84013619

C -1.20688200 -1.01206700 -0.36711519

C 1.20688200 -1.01206700 -0.36711519

H 0.00000000 -2.19217200 -1.68269819

H -2.14722600 -1.35860600 -0.78246119

H 2.14722600 -1.35860600 -0.78246119

H 0.00000000 -0.88594600 2.24468781

H -0.00000000 0.88594600 2.24468781

GS*C_s_* C_9_H_10_ (**9**)

C -0.45844810 1.32627415 0.00000000

C 0.44382246 1.13376310 1.19250300

C 0.81979857 -0.10820951 1.52765500

C -1.56604482 0.25713832 0.00000000

C 0.31876617 -1.26808897 0.78732800

C -0.95044136 -1.11516730 0.00000000

H -0.90222472 2.32530540 0.00000000

H 0.84265349 2.00236557 1.70943200

H 1.53158080 -0.27754656 2.33155500

H 0.52170340 -2.24456654 1.21512300

H -1.63315683 -1.96009904 0.00000000

C 0.81979857 -0.10820951 -1.52765500

C 0.44382246 1.13376310 -1.19250300

C 0.31876617 -1.26808897 -0.78732800

H 1.53158080 -0.27754656 -2.33155500

H 0.84265349 2.00236557 -1.70943200

H 0.52170340 -2.24456654 -1.21512300

H -2.19776733 0.38762122 -0.88581700

H -2.19776733 0.38762122 0.88581700

GS *C_s_* C_8_BH_9_ (**10**)

C -0.57587039 1.36547574 0.00000000

C 0.35102737 1.16336498 1.18876300

C 0.77366310 -0.06496333 1.50791800

C 0.32551056 -1.25438393 0.76570500

C -0.99960767 -1.19569172 0.00000000

H -1.00527718 2.36871871 0.00000000

H 0.73258162 2.03943069 1.70718800

H 1.51893431 -0.20681755 2.28611800

H 0.55747465 -2.21553022 1.21476500

H -1.57582028 -2.11533626 0.00000000

C 0.77366310 -0.06496333 -1.50791800

C 0.35102737 1.16336498 -1.18876300

C 0.32551056 -1.25438393 -0.76570500

H 1.51893431 -0.20681755 -2.28611800

H 0.73258162 2.03943069 -1.70718800

H 0.55747465 -2.21553022 -1.21476500

B -1.63405176 0.19505792 0.00000000

H -2.81616890 0.39024544 0.00000000

TS *C*_2_*_v_* C_8_BH_9_ (**11**)

C 0.00000000 1.29283800 0.82459910

C 0.96148300 1.21041700 -0.33327590

C 1.46689600 -0.00000000 -0.78900390

C 0.96148300 -1.21041700 -0.33327590

C 0.00000000 -1.29283800 0.82459910

H 0.00000000 2.25829100 1.32723310

H 1.31063100 2.14605200 -0.75759090

H 2.11838800 -0.00000000 -1.65800390

H 1.31063100 -2.14605200 -0.75759090

H 0.00000000 -2.25829100 1.32723310

C -1.46689600 -0.00000000 -0.78900390

C -0.96148300 1.21041700 -0.33327590

C -0.96148300 -1.21041700 -0.33327590

H -2.11838800 -0.00000000 -1.65800390

H -1.31063100 2.14605200 -0.75759090

H -1.31063100 -2.14605200 -0.75759090

B 0.00000000 -0.00000000 1.67764310

H 0.00000000 -0.00000000 2.87516910

GS*C_s_* C_8_BH_9_ (**12**)

C -0.57587039 1.36547574 0.00000000

C 0.35102737 1.16336498 0.76500000

C 0.77366310 -0.06496333 1.50791800

C 0.32551056 -1.25438393 1.18875000

C -0.99960767 -1.19569172 0.00000000

H -1.00527718 2.36871871 0.00000000

H 0.73258162 2.03943069 1.28342500

H 1.51893431 -0.20681755 2.28611800

H 0.55747465 -2.21553022 1.63781000

H -1.57582028 -2.11533626 0.00000000

C 0.77366310 -0.06496333 -1.50791800

C 0.35102737 1.16336498 -0.76500000

C 0.32551056 -1.25438393 -1.18875000

H 1.51893431 -0.20681755 -2.28611800

H 0.73258162 2.03943069 -1.28342500

H 0.55747465 -2.21553022 -1.63781000

B -1.63405176 0.19505792 0.00000000

H -2.81616890 0.39024544 0.00000000
